# Fatal heart disease in patients with bone and soft tissue sarcoma

**DOI:** 10.3389/fcvm.2022.951940

**Published:** 2022-10-13

**Authors:** Bei Chen, Xin Zhao, Xiying Li, Jun Liu, Juyu Tang

**Affiliations:** ^1^Department of Orthopaedics, Xiangya Hospital, Central South University, Changsha, China; ^2^Department of Musculoskeletal Oncology, Chenzhou No. 1 People’s Hospital, Chenzhou, China; ^3^Department of Hand and Microsurgery, Xiangya Hospital, Central South University, Changsha, China

**Keywords:** sarcoma, chemotherapy, osteosarcoma, heart diseases, standardized mortality ratio

## Abstract

**Background/purpose:**

With improved cancer survivorship, non-cancer events, especially heart disease (HD), have become the underlying cause of death in cancer patients, but the risk of HD mortality in sarcoma patients remains poorly characterized. Therefore, our purpose was to: (1) identify sarcoma patients at the highest risk of fatal HD compared with the general population, (2) identify patients and sarcoma characteristics associated with a higher risk of HD death, and (3) determine if chemotherapy increased the risk of HD death in sarcoma patients.

**Methods:**

From 1975 to 2016, we identified patients diagnosed with bone and soft tissue sarcoma from the Surveillance, Epidemiology, and End Results (SEER) database in the US. Standardized mortality ratios (SMRs) were evaluated using mortality data from the general population collected by the National Center for Health Statistics. This was the largest retrospective cohort study of fatal HD in individuals with sarcoma.

**Results:**

In 80,905 sarcoma patients observed for 530,290 person-years, 3,350 deaths from HD were identified with a mortality of 631.7/100,000 person-years. The SMR of death from HD was 1.38 (95% CI: 1.33–1.42). The highest risks of death from HD were observed in patients with Ewing sarcoma (SMR = 5.44; 95% CI: 3.38–8.75) and osteosarcoma (SMR = 1.92; 95% CI: 1.55–2.38). Patients diagnosed at < 19 years old had the highest SMR in all age subgroups, and a higher risk of fatal HD relative to the general population was observed in sarcoma survivors diagnosed at < 85 years old. In patients diagnosed at < 19 years old, HD plurality occurred in those with Ewing sarcoma (29.4%) and osteosarcoma (32.4%) and at > 35 years old, HD plurality occurred in those diagnosed with liposarcoma (19.0%) and malignant fibro histiocytoma (MFH) (23.6%). For sarcoma survivors, HD mortality risks were highest within the first year after diagnosis (SMR = 1.31; 95% CI: 1.21–1.41), and this risk remained elevated throughout follow-up compared with the general population. Subgroup analyses indicated that chemotherapy significantly increased the risk of fatal HD in patients with localized osteosarcoma (Hazard ratio (HR) = 3.18; 95% CI: 1.24–8.13; P = 0.016), but not in patients with other histological sarcoma subtypes and clinical stages.

**Conclusion:**

The risk of death from HD mainly varied in patients with different histological sarcoma subtypes and clinical stages. Chemotherapy increased the risk of fatal HD in patients with localized osteosarcoma. To lower the risk of fatal HD in patients with sarcoma, we call for enhanced multidisciplinary cooperation, including cardiologists and orthopedic surgeons.

## Introduction

Over several decades, heart diseases (HD) have become the first leading cause of death globally, and in 2019, killed approximately nine million individuals, accounting for 9% of all deaths (1). Cancer and HD may occur separately in patients, or cancer may cause HD via non-bacterial thrombotic endocarditis and chemoradiation therapy (2–4). As survival rate of cancer patients improve, medical officers have gradually realized that the risk of death from other non-cancer diseases in cancer patients was higher when compared with the general population (5–7), furthermore, HD as the first leading cause of non-cancer death among cancer patients have attracted more and more attention from clinicians (7–9).

Bone and soft tissue sarcoma comprise several rare malignant tumors which arise from mesenchymal tissue (10), they are responsible for more deaths than testicular cancer, Hodgkin’s disease, and thyroid cancer combined due to their more recurrent and metastatic nature (11). The standard therapy for patients with sarcoma is neoadjuvant chemotherapy, surgical resection, and adjuvant chemotherapy (12). Anthracyclines are commonly used chemotherapy drugs, but may cause HD, reduce the quality of life in patients, and increase mortality in sarcoma patients (3). Previous studies reporting the risk of HD in sarcoma patients were limited by small sample sizes and data collected primarily at single institutions (13–16). Currently, limited guidelines are available on fatal HD prevention, identification, or management, specifically in sarcoma patients. One strategy aimed at preventing fatal HD in this group is to identify and target subgroups at greatest HD risk, therefore, retrospective cohort studies such as ours could be used by clinicians to generate survivorship programs and mitigate HD risks in these patients.

In our study, we had the following objectives: (1) to identify sarcoma patients at highest risk of fatal HD compared with the general population, (2) to identify patients and sarcoma characteristics associated with a higher risk of HD death, and (3) to determine if chemotherapy increased the risk of HD death in sarcoma patients.

## Materials and methods

**FIGURE 1:**
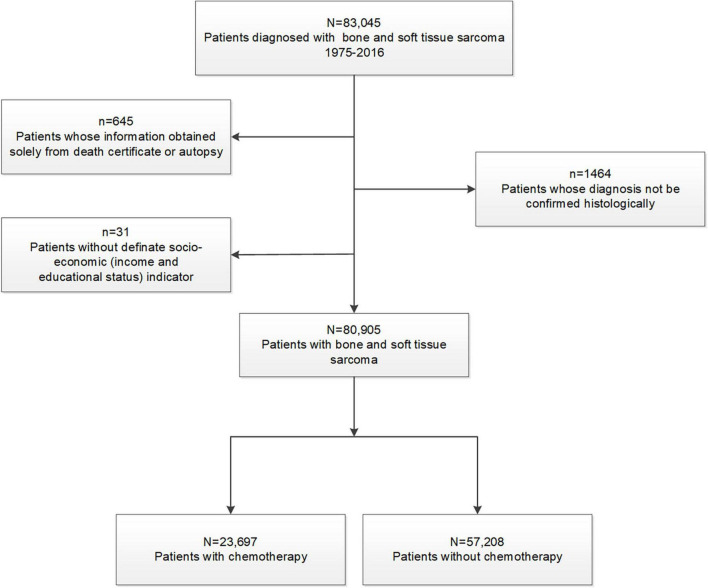
Flowchart describing initial dataset and exclusions leading to final study cohorts.

In this retrospective cohort study, we used the Surveillance, Epidemiology, and End Results (SEER) database at the National Cancer Institute, which comprised 18 registries and covered approximately 28% of the US general population (17). Given that anthracyclines or cisplatin were discovered in the 1980s, we included patients diagnosed with sarcoma between 1975 and 2016 (18). As a comparison, general population mortality data, spanning 1969–2016 from the National Center for Health Statistics, were used (5, 17, 19). Using exclusion criteria (Figure 1), we identified the final study cohort. Patients whose information was obtained solely from death certificates or autopsies were excluded due to no survival time data (<1.5% of patients). Patients diagnosed with bone and soft tissue sarcoma without a definite socioeconomic status were also excluded (11). Sarcomas were classified into ten histological subtypes according to the International Classification of Disease for Oncology third revision (ICD-O-3), and included chondrosarcoma, osteosarcoma, Ewing sarcoma, liposarcoma, malignant fibro histiocytoma (MFH), leiomyosarcoma, fibrosarcoma, synovial sarcoma, Malignant Peripheral Nerve Sheath Tumor (MPNST), and others (ICD-O-3 codes are shown in Table 1) (11).

**TABLE 1:** Death from HD among patients with bone and soft tissue sarcomas by demographic and tumor characteristics.

Characteristic	Patients with cancer in SEER		HD death		Person-years accrued	Mortality^1^	SMR^2^	95% CI
						
	No.	%	No.	%				
Sex								
Female	36283	45	1353	40	247636	546.4	1.45	1.37–1.53
Male	44622	55	1997	60	282655	706.5	1.33	1.28–1.39
Race^3^								
White	65618	81	2876	84	433078	664.1	1.35	1.30–1.40
Black	8530	11	303	9	54297	558.0	1.48	1.32–1.66
Other	6757	8	171	5	42915	398.5	1.70	1.47–1.98
American Indian/Alaska Native	553	1	12	1	3762	318.9	2.13	1.21–3.75
Asian or Pacific Islander	5430	7	149	4	33934	439.1	1.70	1.45–2.00
Unknown	774	1	10	1	5218	191.6	1.42	0.77–2.64
Year of diagnosis								
1975–1977	1667	2	194	6	22941	845.6	1.75	1.52–2.02
1978–1980	1797	2	183	5	25057	730.3	1.86	1.61–2.15
1981–1983	1879	2	184	5	25039	734.8	2.00	1.73–2.31
1984–1986	1986	2	180	5	25146	715.8	1.73	1.50–2.00
1987–1989	2055	3	180	5	26061	690.7	1.61	1.39–1.86
1990–1992	2683	3	206	6	31502	653.9	1.65	1.44–1.89
1993–1995	3593	4	237	7	38861	609.9	1.43	1.26–1.62
1996–1998	3984	5	251	7	40156	625.1	1.37	1.21–1.55
1999–2001	7253	9	376	11	63231	594.6	1.37	1.24–1.52
2002–2004	9463	12	372	11	71767	518.3	1.09	0.98–1.20
2005–2007	9997	12	347	10	60811	570.6	1.14	1.03–1.27
2008–2011	14594	18	387	12	64184	603.0	1.19	1.08–1.32
2012–2016	19954	25	253	8	35529	712.1	1.29	1.14–1.46
Marital status								
Married	38650	48	1690	50	257737	655.7	1.24	1.18–1.30
Unknown	4312	5	233	7	26155	890.8	1.57	1.38–1.78
Unmarried	37943	47	1427	43	246397	579.1	1.54	1.47–1.63
Education^4^								
High	26221	32	888	27	137715	644.8	1.39	1.30–1.49
Median	27786	34	1227	37	190351	644.6	1.38	1.30–1.45
Low	26898	33	1235	37	202224	610.7	1.37	1.29–1.44
Income^4^								
High	26657	33	1159	35	191456	605.4	1.32	1.24–1.39
Median	23844	29	910	27	156387	581.9	1.28	1.20–1.37
Low	30404	38	1281	38	182447	702.1	1.52	1.44–1.61
Insurance								
Uninsured	1259	2	13	1	3741	347.5	2.25	1.31–3.88
Any Medicaid	5668	7	73	2	15481	471.5	2.13	1.69–2.68
Insured	29498	36	621	19	95223	652.1	1.13	1.04–1.22
Unknown	44480	55	2643	78	415844	635.6	1.44	1.38–1.49
Histology^5^								
Ewing sarcoma	3055	4	17	1	21430	79.3	5.44	3.38–8.75
Osteosarcoma	6504	8	83	2	46552	178.3	1.92	1.55–2.38
Synovial sarcoma	3171	4	42	1	23181	181.2	1.55	1.14–2.10
MPNST	2116	3	64	2	11374	562.7	1.57	1.23–2.00
Chondrosarcoma	5941	7	232	7	50229	461.9	1.37	1.20–1.55
MFH	8492	10	775	23	66089	1172.7	1.34	1.25–1.44
Fibrosarcoma	4102	5	185	6	29838	620.0	1.27	1.10–1.47
Liposarcoma	10819	13	623	19	81894	760.7	1.26	1.16–1.36
Leiomyosarcoma	7532	9	338	10	41938	805.9	1.18	1.06–1.31
Chordoma	1605	2	55	2	10147	542.0	1.05	0.81–1.37
Others	27568	34	936	28	147614	634.1	1.59	1.49–1.69
Grade at presentation^6^								
Grade I	9595	12	485	14	84323	575.2	1.22	1.11–1.33
Grade II	10074	12	438	13	80079	547.0	1.23	1.12–1.35
Grade III	11701	14	429	13	58801	729.6	1.39	1.27–1.53
Grade IV	17159	21	559	17	76839	727.5	1.34	1.24–1.46
Unknown	32376	40	1439	43	230247	625.0	1.51	1.43–1.59
Stage at presentation								
Localized	42042	52	2152	64	332076	648.0	1.33	1.27–1.38
Regional	19671	24	706	21	126953	556.1	1.32	1.23–1.42
Distant	12414	15	199	6	30812	645.8	2.11	1.84–2.42
Unknown	6778	8	293	9	40448	724.4	1.60	1.42–1.79
Chemotherapy								
No/Unknown	57208	71	3059	91	395330	773.8	1.35	1.31–1.40
Yes	23697	29	291	9	134960	215.6	1.69	1.51–1.90
Radiotherapy								
No/Unknown	52386	65	2314	69	355007	651.8	1.49	1.43–1.56
Yes	28519	35	1036	31	175283	591.0	1.17	1.10–1.25
Surgery								
None	14481	18	427	13	40693	1049.3	2.16	1.96–2.37
Yes	64412	80	2826	84	476861	592.6	1.29	1.24–1.34
Unknown	2012	2	97	3	12736	761.6	2.04	1.67–2.49
All sarcoma patients	80905	100	3350	100	530290	631.7	1.38	1.33–1.42

HD, heart diseases; SEER, Surveillance, Epidemiology, and End Results; SMR, standardized mortality ratio; MFH, malignant fibrohistiocytoma; MPNST, Malignant Peripheral Nerve Sheath Tumor; NOS, not of specific.

^1^Per 100,000 person-years.

^2^SMRs were estimated as the ratios of observed to expected number of deaths. Observed number of HD death represent the total number of deaths from HD among patients with sarcoma recorded during the study period. Expected death represent the number of individuals who died of HD in the general population with a similar distribution of age at diagnosis (5-year intervals), sex, race (white, black, and other), and calendar year of diagnosis (3-year intervals). The calculation for SMRs were adjusted to the age, sex, race/ethnicity, and calendar year distributions between sarcoma patients and the general population.

^3^Others included American Indian/AK Native, Asian/Pacific Islander and unknown race.

^4^Education status and income level were categorized into tertiles.

^5^ICD-O-3: Chondrosarcoma, 9220–9243; Osteosarcoma, 9180–9200; Ewing sarcoma, 9260; Liposarcoma, 8850–8858; MFH, 8830; Leiomyosarcoma, 8890–8891 and 8896; Synovial sarcoma, 9040–9044; MPNST, 9540 and 9561; Chordoma, 9370–9372.

^6^Grade I: Well differentiated; Grade II: Moderately differentiated; Grade III: Poorly differentiated; Grade IV: Undifferentiated; anaplastic.

We extracted demographic data, including age at diagnosis, sex (female, male), race (white, black, and others), calendar year of diagnosis (1975–2016), marital status at diagnosis (married, unmarried, and unknown), insurance status (insured, Medicaid, uninsured, unknown), and socioeconomic indicators (income and educational status). Income (median family income) and educational level (percentage of individuals > 25 years of age with at least a high school degree) from county-level data were calculated by referring to 2000 US census data and categorizing data into tertiles (high, median, low). Tumor-related characteristics included histological subtype, grade (I–IV), clinical sarcoma stage (localized, regional, distant, and unknown), and treatment information (chemotherapy, radiotherapy, and surgery status). Time of follow-up and cause of death were also available and collected. Patients were considered to have committed HD death if the cause of death variable was coded as “Diseases of Heart (50060).”

For objective 1, we calculated standardized mortality ratios (SMRs), which provided the relative risk of HD death for sarcoma patients when compared with all US residents, stratified by histological subgroup. SMRs and 95% confidence intervals (CIs) were calculated as previously described (19, 20). Briefly, SMRs were estimated as the ratio of observed to expected numbers of deaths. The observed number of HD deaths represented the total number of deaths from heart events in sarcoma patients during the follow-up period; the expected number of deaths represented the number of deaths from heart events in the general population with a similar age at diagnosis, sex, race, and calendar year distribution. Five-year age categories and three-year calendar categories were used for standardization (21). Mortality from fatal HD was calculated as the number of deaths from HD divided by person-years at risk (20).

For objective 2, considering other competitive risk events, we plotted cumulative incidence curves and compared them using the Gray test. Furthermore, we constructed logistic regression and multivariate Cox proportional hazards models to identify risk factors associated with a higher risk of HD death in sarcoma patients.

For objective 3, we conducted subgroup analysis by histological subtype and clinical stage to determine if chemotherapy increased the risk of fatal HD in sarcoma patients. The survival time was from sarcoma diagnosis until fatal HD; values recorded as 0 months in the SEER database were converted to one-half of a month according to accepted epidemiological practices (19). Statistical significance was accepted at *P* < 0.05 (two-sided). Analyses were performed using SEER*Stat software version 8.3.6 and R version 3.51 statistical software.

## Results

In total, 3,350 HD deaths were identified in 80,905 patients with bone and soft tissue sarcoma over 530,291 person-years, and provided an age-, sex-, race-, and year-adjusted HD mortality of 631.7/100,000 person-years. The corresponding HD mortality in the general US population was 16.7/100,000 person-years. This generated an SMR = 1.38 (95% CI: 1.33–1.42). The survival time range was 0–39.25 years, with a mean survival time of 7.34 years for sarcoma patients dying from HD.

### Sarcoma patient risk of fatal heart disease versus the general population

**FIGURE 2:**
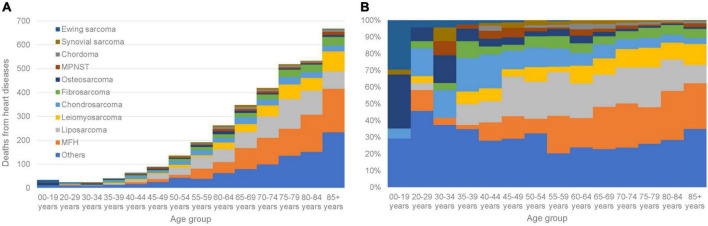
Fatal HD among sarcoma patients as a function of age group. **(A)** The *y*-axis depicts the absolute number of HD deaths and the *x*-axis depicts the age group at time of diagnosis. The colors depict the histological subtypes of sarcoma. **(B)** The *y*-axis depicts the relative number of HD deaths and the *x*-axis depicts the age group at time of diagnosis. The colors depict the histological subtypes of sarcoma.

The characteristics of sarcoma patients and those who died of HD vs. all cancer patients are shown (Table 1). Higher SMRs for fatal HD in patients with sarcoma were associated with the female sex (546.4/100,000 person-years; SMR = 1.45; 95% CI: 1.37–1.53), the American Indian/Alaskan Native race (318.9/100,000 person-years; SMR = 2.13; 95% CI: 1.21–3.75), and an unmarried status (579.1/100,000 person-years; SMR = 1.54; 95% CI: 1.47–1.63). For patients as a whole, the risk of fatal HD was higher when compared with the general population over the 40 years covered in the SEER data, except for those diagnosed between 2002 and 2004 (518.3/100,000 person-years; SMR = 1.09; 95% CI: 0.98–1.20). Patients with high and low educational levels were equal likely to die from HD: 32% vs. 33%. Patients with low income levels (SMR = 1.52; 95% CI: 1.44–1.61) and an uninsured status (SMR = 2.25; 95% CI: 1.31–3.88) had a higher SMR for fatal HD when compared with those with a high income level (SMR = 1.32; 95% CI: 1.24–1.39) and an insured status (SMR = 1.13; 95% CI: 1.04–1.22). Patients diagnosed at a younger age had a higher SMR for HD, and SMRs gradually declined as patients were diagnosed at later ages (Table 2); patients < 19 years old had an SMR = 9.46 (95% CI: 6.76–13.24, 32.7/100,000 person-years) vs. >85 years old who had an SMR = 1.04 (95% CI: 0.96–1.12, 7,295.3/100,000 person-years). Fatal HD in sarcoma patients as a function of age group is shown (Figure 2). In patients diagnosed at <19 years old, HD plurality occurred in those with Ewing sarcoma (29.4%) and osteosarcoma (32.4%). In contrast, in patients diagnosed at >35 years old, HD plurality occurred in those diagnosed with liposarcoma (19.0%) and MFH (23.6%).

**TABLE 2:** Risk of fatal HD among patients with sarcoma by age at diagnosis.

Age at diagnosis (years)	Mortality in general population^1^	Mortality in patients with sarcoma^2^	Person-years accrued	No. of deaths	SMR^3^	95% CI
00–19	2.6	32.7	103,970	34	9.46	6.76–13.24
20–29	4.5	42.7	56,215	24	7.24	4.86–10.81
30–34	7.7	76.9	31,216	24	5.59	3.75–8.34
35–39	14.5	113.7	35,173	40	4.11	3.01–5.60
40–44	29.2	170.1	37,626	64	3.09	2.42–3.95
45–49	58.8	217.6	40,893	89	2.17	1.77–2.68
50–54	109.5	330.0	41,213	136	1.96	1.65–2.32
55–59	188.7	481.2	39,694	191	1.71	1.49–1.97
60–64	310.4	709.5	36,925	262	1.60	1.41–1.80
65–69	508.0	1,050.7	33,121	348	1.55	1.40–1.72
70–74	804.0	1,501.6	27,903	419	1.37	1.24–1.51
75–79	1308.2	2,303.0	22,535	519	1.31	1.20–1.43
80–84	2133.1	3,636.1	14,658	533	1.22	1.12–1.33
85+	3586.2	7,295.3	9,142	667	1.04	0.96–1.12

HD, heart diseases; SMR, standardized mortality ratio.

^1^Per 100,000 person-years. Reference population: general US population, 1969–2016.

^2^Among persons with sarcoma in the populations served by the SEER program.

^3^Adjusted to the race and sex distributions between patients and the general population.

### Histological sarcoma subtype is associated with a higher risk of fatal heart disease

The risk of fatal HD in patients with most sarcoma subtypes was higher when compared with the general US population, except for chordoma (542.0/100,000 person-years; SMR = 1.05; 95% CI: 0.81–1.37) (Table 1). The highest SMR was observed in patients with Ewing sarcoma (79.3/100,000 person-years; SMR = 5.44; 95% CI: 3.38–5.75), followed by osteosarcoma (178.3/100,000 person-years; SMR = 1.92; 95% CI: 1.55–2.38), MPNST (562.7/100,000 person-years; SMR = 1.57; 95% CI: 1.23–2.00), and synovial sarcoma (181.2/100,000 person-years; SMR = 1.55; 95% CI: 1.14–2.10). Also, patients with MFH had the highest mortality for fatal HD across all patients (1172.7/100,000 person-years; SMR = 1.34; 95% CI: 1.25–1.44). In patients with Ewing sarcoma, osteosarcoma, MPNST, and MFH with chemotherapy a higher relative risk of fatal HD was observed when compared with the general population (Table 3). However, patients with synovial sarcoma (SMR = 1.69; 95% CI: 0.88–3.52), chondrosarcoma (SMR = 1.10; 95% CI: 0.52–2.30), fibrosarcoma (SMR = 1.28; 95% CI: 0.64–2.56), liposarcoma (SMR = 1.25; 95% CI: 0.83–1.88), leiomyosarcoma (SMR = 1.30; 95% CI: 0.87–1.94), and chordoma (SMR = 0.66; 95% CI: 0.09–4.67), and receiving chemotherapy had an equal risk when compared with the general population.

**TABLE 3:** Standardized mortality ratios (SMRs) of fatal HD among patients with sarcoma by histology and chemotherapy status.

Histology	Patients without chemotherapy					Patients with chemotherapy				
	No. of deaths	No. of patients	Person-years	Mortality^1^	SMR (95% CI)	No. of deaths	No. of patients	Person-years	Mortality^1^	SMR (95% CI)
Ewing sarcoma	3	241	1,796	167.0	5.12 (1.65–15.87)	14	2,814	19,634	71.3	5.52 (3.27–9.31)
Osteosarcoma	45	1,683	11,899	378.2	1.49 (1.11–1.99)	38	4,821	34,654	109.7	2.93 (2.13–4.02)
Synovial sarcoma	33	1,797	14,677	224.8	1.51 (1.08–2.13)	9	1,374	8,504	105.8	1.69 (0.88–3.25)
MPNST	57	1,591	9,338	610.4	1.47 (1.33–1.90)	7	525	2,037	343.7	3.59 (1.71–7.52)
Chondrosarcoma	225	5,352	47,285	475.8	1.38 (1.21–1.57)	7	589	2,944	237.8	1.10 (0.52–2.30)
MFH	716	6,973	56,245	1,273.0	1.33 (1.24–1.43)	59	1,519	9,845	599.3	1.41 (1.09–1.82)
Fibrosarcoma	177	3,613	27,338	647.4	1.27 (1.10–1.48)	8	489	2,500	320.0	1.28 (0.64–2.56)
Liposarcoma	600	9,735	75,766	791.9	1.26 (1.16–1.36)	23	1,084	6,129	375.3	1.25 (0.83–1.88)
Leiomyosarcoma	314	5,997	36,725	855.0	1.17 (1.05–1.31)	24	1,535	5,214	460.3	1.30 (0.87–1.94)
Chordoma	54	1,532	9,782	552.0	1.06 (0.81–1.39)	1	73	366	273.3	0.66 (0.09–4.67)
Others	835	18,694	104,478	799.2	1.56 (1.46–1.67)	101	8,874	43,136	234.1	1.80 (1.48–2.18)

SMR, standardized mortality ratio; HD, heart diseases; MFH, Malignant fibrohistiocytoma; MPNST, Malignant Peripheral Nerve Sheath Tumor.

^1^Per 100,000 person-years.

### Risk of heart disease death over time after diagnosis

For all sarcoma subtypes, the risk of death from HD was higher relative to the general population in the first year after a diagnosis, but decreased gradually from 1–5 years, but then increased after this (Table 4). The relative risk of fatal HD in sarcoma patients when compared with the general population was highest in the 10 years after a sarcoma diagnosis (SMR = 2.85; 95% CI: 2.68–3.04). For most sarcoma types, the risk of death from HD was equal to the general population in the first year of diagnosis. For patients with osteosarcoma, the relative risk was higher when compared with the general population in the first year after a sarcoma diagnosis (SMR = 1.73; 95% CI: 1.12–2.69) and after 10 years (SMR = 4.75; 95% CI: 3.43–6.59).

**TABLE 4:** Fatal HD among patients with sarcoma by histological subtypes and years since diagnosis.

Histology	Time since diagnosis			
	0–1 Year	1–5 Years	5–10 Years	>10 Years
**All**				
No. of deaths	675	1016	720	939
Person-years	69,322	181,220	130,829	154,069
SMR^1^	1.31	0.94	1.32	2.85
95%CI	1.21–1.41	0.89–1.00	1.22–1.42	2.68–3.04
Chondrosarcoma				
No. of deaths	27	73	38	94
Person-years	5,388	16,056	12,997	16,202
SMR	0.9	1.05	0.92	2.98
95%CI	0.62–1.31	0.84–1.33	0.67–1.27	2.44–3.65
**Chordoma**				
No. of deaths	9	16	17	13
Person-years	1,458	4,184	2,643	1,973
SMR	0.84	0.61	1.48	2.77
95%CI	0.44–1.62	0.37–0.99	0.92–2.38	1.61–4.78
**Ewing sarcoma**				
No. of deaths	1	4	3	9
Person-years	2,804	7,044	4,947	6,844
SMR	1.28	3.42	4.81	15.16
95%CI	0.18–9.09	1.28–9.11	1.55–14.91	7.89–29.13
**Fibrosarcoma**				
No. of deaths	33	62	38	52
Person-years	3,685	10,331	7,218	8,885
SMR	1.1	0.91	1.17	3.12
95%CI	0.78–1.54	0.71–1.17	0.85–1.61	2.38–4.1
**Leiomyosarcoma**				
No. of deaths	72	115	77	74
Person-years	6,450	16,327	10,387	9,252
SMR	1.13	0.86	1.25	2.33
95%CI	0.89–1.42	0.72–1.03	1.00–1.57	1.86–2.93
**Liposarcoma**				
No. of deaths	72	153	162	236
Person-years	9,812	28,926	21,418	22,498
SMR	0.88	0.73	1.28	2.89
95%CI	0.70–1.11	0.62–0.85	1.10–1.49	2.54–3.28
**MFH**				
No. of deaths	120	250	184	221
Person-years	7,472	20,681	16,734	21,766
SMR	1.06	0.99	1.35	2.60
95%CI	0.89–1.27	0.87–1.12	1.17–1.56	2.28–2.97
**MPNST**				
No. of deaths	14	19	12	19
Person-years	1,805	4,148	2,872	2,677
SMR	1.62	1.02	1.32	3.8
95%CI	0.96–2.74	0.65–1.60	0.75–2.32	2.43–5.96
**Osteosarcoma**				
No. of deaths	20	15	12	36
Person-years	5,743	14,378	10,884	15,972
SMR	1.73	0.96	1.33	4.75
95%CI	1.12–2.69	0.58–1.59	0.76–2.35	3.43–6.59
**Synovial sarcoma**				
No. of deaths	8	10	7	17
Person-years	2,872	7,842	5,681	7,005
SMR	1.46	0.93	1.12	3.35
95%CI	0.73–2.91	0.50–1.74	0.53–2.35	2.08–5.39
**Others**				
No. of deaths	299	299	170	168
Person-years	21,827	51,298	35,044	40,989
SMR	1.87	1.12	1.51	2.82
95%CI	1.67–2.09	1.00–1.25	1.30–1.76	2.43–3.28

SMR, standardized mortality ratio; MFH, Malignant fibrohistiocytoma; MPNST, Malignant Peripheral Nerve Sheath Tumor; NOS, not of specific.

^1^Reference population: general US population, 1969 to 2016. Adjusted to the age, sex and race distributions between patients and the general population.

### Characteristics associated with a higher risk of fatal heart disease

From multivariable logistic regression (Table 5) of sarcoma patients, older age at diagnosis [odds ratio (OR) = 1.06; 95% CI: 1.05–1.07; *P* < 0.001], male sex (OR = 1.44; 95% CI: 1.33–1.56; *P* < 0.001), black race (OR = 1.28; 95% CI: 1.12–1.45; *P* < 0.001), unmarried status (OR = 1.22; 95% CI: 1.13–1.33; *P* < 0.001), and a low income (OR = 1.16; 95% CI: 1.05–1.29; *P* = 0.004) were associated with significantly greater odds of dying from HD. Neither educational level nor insurance status were associated with a risk of dying from HD in the multivariable model. Moreover, sarcoma patients with distant metastases of sarcoma had a lower OR of fatal HD than patients with localized sarcoma (OR = 0.36; 95% CI: 0.3–0.43; *P* < 0.001). Receiving chemotherapy was associated with marginally lower odds of fatal HD (vs. without chemotherapy; OR = 0.77; 95% CI: 0.67–0.88; *P* < 0.001). As shown (Table 5 – Cox proportional hazards model in the right panel), the HRs of patients who died of HD are stratified by subgroup. In the Cox regression model, receiving radiotherapy (vs. without radiotherapy; HR = 0.80; 95% CI: 0.74–0.87; *P* < 0.001) and surgery (vs. without surgery; HR = 0.56; 95% CI: 0.50–0.63; *P* < 0.001) was still associated with a lower odds of fatal HD.

**TABLE 5:** Odds ratios and hazard ratios of fatal HD among sarcoma patients.

	Logistic regression model			Cox proportional hazards model		
	Odds ratio	95% CI	*P*-value	Hazard ratio	95% CI	*P*-value
Age at diagnosis^1^	1.06	1.05–1.07	<0.001	1.10	1.09–1.12	<0.001
Sex						
Female	Reference			Reference		
Male	1.44	1.33–1.56	<0.001	1.63	1.51–1.75	<0.001
Race						
White	Reference			Reference		
Black	1.27	1.11–1.45	<0.001	1.33	1.18–1.50	<0.001
Other	0.83	0.70–0.97	0.025	0.79	0.68–0.93	0.003
Year of diagnosis^1^	0.94	0.92–0.95	<0.001	0.97	0.96–0.98	<0.001
Marital status						
Married	Reference			Reference		
Unmarried	1.22	1.13–1.33	<0.001	1.48	1.37–1.60	<0.001
Unknown	1.33	1.14–1.55	<0.001	1.27	1.11–1.47	0.001
Income						
High	Reference			Reference		
Medium	1.00	0.91–1.09	0.922	1.02	0.94–1.12	0.592
Low	1.16	1.05–1.29	0.004	1.22	1.11–1.34	<0.001
Education						
High	Reference			Reference		
Medium	0.96	0.86–1.07	0.428	0.97	0.88–1.07	0.542
Low	0.99	0.88–1.11	0.829	0.99	0.89–1.11	0.894
Insurance						
Uninsured	Reference			Reference		
Any Medicaid	1.03	0.58–1.96	0.923	0.94	0.70–2.14	0.831
Insured	0.85	0.51–1.56	0.566	0.59	0.34–1.02	0.060
Unknown	1.27	0.76–2.34	0.399	0.59	0.34–1.03	0.062
Grade at presentation						
Grade I	Reference			Reference		
Grade II	0.90	0.78–1.03	0.127	1.07	0.94–1.22	0.312
Grade III	0.83	0.72–0.96	0.010	1.41	1.23–1.61	<0.001
Grade IV	0.78	0.68–0.89	<0.001	1.35	1.19–1.54	<0.001
Other	0.84	0.75–0.94	0.003	1.21	1.09–1.35	<0.001
Stage at presentation						
Localized	Reference			Reference		
Regional	0.76	0.69–0.84	<0.001	1.02	0.94–1.12	0.589
Distant	0.36	0.31–0.43	<0.001	1.31	1.12–1.53	<0.001
Unknown	0.70	0.61–0.81	<0.001	0.91	0.80–1.04	0.156
Radiotherapy						
No/Unknown	Reference			Reference		
Yes	0.84	0.77–0.91	<0.001	0.80	0.74–0.87	<0.001
Chemotherapy						
No/Unknown	Reference			Reference		
Yes	0.77	0.67–0.88	<0.001	0.97	0.85–1.11	0.659
Surgery						
None	Reference			Reference		
Yes	1.18	1.04–1.33	0.009	0.56	0.50–0.63	<0.001
Unknown	0.53	0.41–0.67	<0.001	0.86	0.63–1.00	0.050

HD, heart diseases; NOS, not of specific.

^1^Increase 1 year.

### Subgroup analysis of the risk of fatal heart disease

**FIGURE 3:**
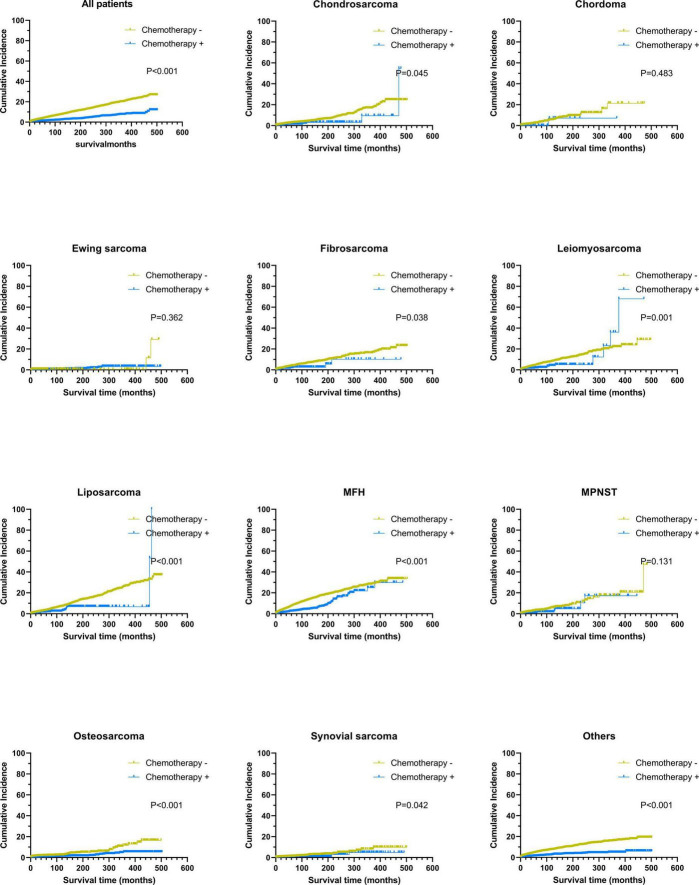
Subgroup analysis: the graph showed cumulative incidence curves of death resulting from heart events. MFH, malignant fibro histiocytoma; MPNST, malignant peripheral nerve sheath tumors.

We conducted subgroup analysis of the study cohort based on histological sarcoma subtypes. Cumulative incidence curves are shown (Figure 3). Survival analysis indicated that all patients with sarcoma benefited from chemotherapy except those with chordoma (*P* = 0.280) and Ewing sarcoma (*P* = 0.555). To further determine if chemotherapy increased the risk of fatal HD in patients with sarcoma, we conducted subgroup analysis by clinical stage. In the subgroup analysis, chemotherapy could protect patients with regional Ewing sarcoma from dying from HD (Table 6). Moreover, we found that chemotherapy could increase significantly the risk of fatal HD among patients with localized osteosarcoma (HR = 3.18; 95% CI: 1.24–8.13; *P* = 0.016), but not those with regional (HR = 0.63; 95% CI: 0.28–1.40; *P* = 0.259) or advanced osteosarcoma (HR = 0.25; 95% CI: 0.06–1.03; *P* = 0.055). For other subgroups with sarcoma at different clinical stages, chemotherapy did not have any effect on the risk of fatal HD.

**TABLE 6:** Subgroup analysis of fatal HD among patients with sarcoma by histology and clinical stage.

Histology	Clinical stage	HR^1^	95% CI	*P*-value
**Ewing sarcoma**				
	All	0.97	0.85–1.11	0.659
	Localized	Inf	0.00 – Inf	0.999
	Regional	0.04	0.01–0.29	0.001
	Distant	0.00	0.00 – Inf	1.000
**Osteosarcoma**				
	All	0.97	0.85–1.11	0.659
	Localized	3.18	1.24–8.13	0.016
	Regional	0.63	0.28–1.40	0.259
	Distant	0.25	0.06–1.03	0.055
**Synovial sarcoma**				
	All	0.97	0.85–1.11	0.659
	Localized	0.40	0.06–2.86	0.363
	Regional	3.06	0.85–11.11	0.088
	Distant	Inf	0.00–Inf	1.000
**MPNST**				
	All	0.97	0.85–1.11	0.659
	Localized	4.59	0.85–24.67	0.076
	Regional	0.71	0.05–10.57	0.804
	Distant	0.87	0.18–4.24	0.867
**Chondrosarcoma**				
	All	0.97	0.85–1.11	0.659
	Localized	0.44	0.10–1.87	0.268
	Regional	1.05	0.30–3.60	0.949
	Distant	0.52	0.10–2.81	0.449
**MFH**				
	All	0.97	0.85–1.11	0.659
	Localized	1.17	0.83–1.65	0.375
	Regional	0.89	0.51–1.53	0.667
	Distant	0.28	0.07–1.16	0.079
**Fibrosarcoma**				
	All	0.97	0.85–1.11	0.659
	Localized	0.94	0.36–2.47	0.902
	Regional	1.76	0.38–8.23	0.474
	Distant	1.06	0.06–18.89	0.968
**Liposarcoma**				
	All	0.97	0.85–1.11	0.659
	Localized	0.84	0.48–1.45	0.523
	Regional	1.33	0.54–3.23	0.534
	Distant	0.49	0.06–3.94	0.504
**Leiomyosarcoma**				
	All	0.97	0.85–1.11	0.659
	Localized	0.83	0.39–1.77	0.635
	Regional	1.31	0.57–2.97	0.524
	Distant	0.44	0.14–1.39	0.162
**Chordoma**				
	All	0.97	0.85–1.11	0.659
	Localized	1.66	0.16–17.36	0.673
	Regional	0.00	0.00 – Inf	0.998
	Distant	0.00	0.00 – Inf	1.000
**Others**				
	All	0.97	0.85–1.11	0.659
	Localized	0.97	0.70–1.34	0.848
	Regional	0.76	0.46–1.24	0.273
	Distant	0.47	0.29–0.77	0.003

HD, heart diseases; HR, hazard ratio; MFH, malignant fibrohistiocytoma; MPNST, Malignant Peripheral Nerve Sheath Tumor.

^1^We performed a survival analysis using a Cox proportional hazards model to calculate hazard ratios (HRs), adjusting for demographics: age, sex, race, year of diagnosis, socioeconomic status, insurance status and tumor characteristics: clinical stage, grade at presentation, radiotherapy and surgery status.

## Discussion

With the development of multimodality therapy, including advances in imaging techniques and neoadjuvant chemotherapy, sarcoma patient survival has improved significantly (22). In addition to prolonging survival rates, clinicians are now increasingly concerned about complications caused by sarcoma therapy (20, 23). Several small sample or single-center clinical cohort studies reported that antineoplastic agents such as anthracyclines increased the risk of dying from HD in sarcoma patients (14, 15). However, due to low sarcoma incidence rates and limitations with small sample or single center studies, no robust data are available to inform clinical practice. To address this, and to our knowledge, ours is the largest retrospective cohort study on the risk of fatal HD in patients with bone and soft tissue sarcoma.

We performed a contemporary analysis of the risk of fatal HD in >80,000 sarcoma patients and showed that HD risk varied as a function of age, histological subtype, clinical stage, and time after diagnosis. Earlier studies reported that cancers were associated with a high risk of death from HD (4, 6, 7). We first reported that sarcoma patients had a higher risk of fatal HD when compared with the general US population, which was potentially attributed to antineoplastic agents such as anthracycline (24). Anthracyclines are antibiotics discovered approximately 50 years ago, are used as antineoplastic agents, and are the most successful anticancer therapies ever developed for sarcoma (3). However, a worrisome adverse side effect is ventricular dysfunction and heart failure (25). Due to low sarcoma incidence rates, previous cardio-toxicity studies in sarcoma patients receiving chemotherapy were conducted in small sample or at single centers (13–15). In a recent comparative study of 95 patients with sarcoma, approximately 17% developed cardio-toxicity after receiving doxorubicin (26). Indeed, the death rate due to HD in patients receiving chemotherapy was seriously underreported due to inaccurate statistics about cause of death.

The systematic and standardized data collection procedures are used to ensure that the causes of death recoded in SEER are accurate (27); therefore, we believe our study presents a reliable picture of death rates from HD in patients with sarcoma in the US. Using the SEER database, we showed that the incidence rate of fatal HD in sarcoma patients was 631.7/100,000 person-years. In subgroup analyses, chemotherapy for sarcoma only increased the risk of fatal HD in patients with localized osteosarcoma, but not for other subtypes. These subgroups results may be attributed to the low incidence rates of Ewing sarcoma in the SEER database.

Apart from chemotherapy, surgery is the main treatment method for sarcoma as it aims to remove all sarcoma traces from patients (22). In 2005, an estimated 18,000 patients underwent amputation due to bone and soft tissue sarcoma, which was the third leading cause of amputation in the US (28). We showed that 79% of patients underwent sarcoma surgery in the study cohort. These patients typically lost a certain degree of physical activity, which is a known risk factor for cardiovascular events (24, 29). Moreover, earlier reports indicated that sarcoma patients presented a certain degree of anxiety and depression which were related to functional and appearance changes (30, 31). Physical inactivity and demoralization are risk factors for HD (32). In our study, the higher relative risk of fatal HD in sarcoma patients to general population in the first year was possibly attributed to chemotherapy, and the relative risk of fatal HD was highest during follow-up after 10 years, which was possibly attributed to combined physical inactivity and demoralization effects. Therefore, clinicians should orchestrate specific rehabilitation care strategies to help long-term sarcoma survivors improve their exercise regimens. Also, evidence now suggests that exercise may inhibit both early and late doxorubicin-induced cardiotoxicity (33). Likewise, psychological assessments such as the Hamilton Depression and Hamilton Anxiety Scales are necessary for long-term sarcoma survivors (20), and can help orthopedic surgeons identify psychological disorders in patients and allow psychologists commence interventions as early as possible.

Cardiovascular events have typically been regarded as age-related disorders, but in our study, younger patients with sarcoma had a higher relative risk of fatal HD when compared with older patients. In the general population aged < 19 years old, HD mortality was very low, at only 2.6/100,000 person-years. But in similar-aged sarcoma patients, this mortality was 32.7/100,000 person-years due to sarcoma and chemotherapy. Given the high risk of fatal HD among younger patients with sarcoma, clinicians should closely monitor cardiac functions in these younger patients.

Our study had several limitations, most of which were related to the SEER database. Firstly, the database program was limited in terms of detailed information on chemoradiotherapy such as doses and cycles. In a phase III ANNOUNCE trial evaluating cardiotoxicity in patients with soft-tissue sarcoma given doxorubicin, cardiac dysfunction occurred in 2% of patients receiving doses of <450 mg/m^2^, 3% at 450 – <600 mg/m^2^, and 1.1% at ≥600 mg/m^2^ (14). Considering the dose-dependent cardiotoxicity of doxorubicin (34, 35), direct associations must be assessed between chemotherapy dose or cycles and the risk of HD death in sarcoma patients. Future analyses can be augmented by the SEER-Medicare data set, which may elucidate direct relationships with chemotherapy doses and cycles. Secondly, in subgroup analyses, we observed no chemotherapy effects on the risk of fatal HD in other subgroups, such as patients with Ewing sarcoma, inconsistent with previous studies (14, 33, 36). A possible explanation could be the relatively low number of deaths due to cardiovascular events which in turn may have reduced the statistical power of our subgroup analyses, therefore, this requires further investigation. Despite these limitations, ours is the first large sample and population-based study with the longest follow-up times, to explore the risk of HD death in sarcoma patients. Our results are reliable and robust and may be used to guide clinical practice.

## Conclusion

This is the first large population-based study on the risk of fatal HD in sarcoma patients, which up to now were rarely reported. Our results suggest that the risk of fatal HD in sarcoma patients is higher than that of the general population, and increases with longer follow-up times. The relative risk of HD to the general population varied in patients with different histological sarcoma subtypes and clinical stage. Subgroup analyses indicated that chemotherapy increased the risk of fatal HD in patients with localized osteosarcoma. To mitigate the risk of fatal HD in sarcoma patients, enhanced multidisciplinary cooperation is warranted, including cardiologists and orthopedic surgeons.

## Data availability statement

The original contributions presented in this study are included in the article/supplementary material, further inquiries can be directed to the corresponding authors.

## Ethics statement

Ethical review and approval was not required for this study in accordance with the local legislation and institutional requirements. Written informed consent from the participants was not required for this study in accordance with the local legislation and institutional requirements.

## Author contributions

BC and JT: conception and design of study. BC and XZ: acquisition of data. BC, XZ, XL, JL, and JT: analysis and/or interpretation of data. BC, XL, JL, and JT: drafting the manuscript. All authors contributed to the article and approved the submitted version.
